# Smartphone and Internet Addictive Behaviors Are Differentially Associated with Mediterranean Diet Adherence in Young Adults: The EVA-Adic Study

**DOI:** 10.3390/nu18152425

**Published:** 2026-07-24

**Authors:** Alberto Vicente-Prieto, Cristina Lugones-Sánchez, Sara Vicente-Gabriel, Cristina Saldaña-Ruiz, Susana González-Sánchez, Sandra Conde-Martín, Manuel A. Gómez-Marcos, Marta Gómez-Sánchez, Leticia Gómez-Sánchez, EVA-Adic Investigators Group

**Affiliations:** 1 Unidad de Investigación en Atención Primaria de Salamanca (APISAL), Gerencia de Atención Primaria de Salamanca, Gerencia Regional de salud de Castilla y León (SACyL), Avenida de Portugal 83, 37005 Salamanca, Spain; albertovicente94@gmail.com (A.V.-P.); cristinals@usal.es (C.L.-S.); u127891@usal.es (S.V.-G.); csaldanar@saludcastillayleon.es (C.S.-R.); gongar04@gmail.com (S.G.-S.); sandraconde@usal.es (S.C.-M.); 2 Instituto de Investigación Biomédica de Salamanca (IBSAL), Paseo de San Vicente, 58-182, 37007 Salamanca, Spain; 3 Departamento de Medicina, Facultad de Medicina, Universidad de Salamanca, Calle Alfonso X el Sabio s/n, 37007 Salamanca, Spain; 4 Red de Investigación en Cronicidad, Atención Primaria y Promoción de la Salud (RICAPPS), Avenida de Portugal 83, 37005 Salamanca, Spain; 5 Servicio de Hospitalización Domiciliaria, Marqués de Valdecilla Hospital Universitario, 39008 Santander, Spain; martagmzsnchz@gmail.com; 6 Servicio de Emergencias, Hospital Universitario de La Paz, P. de la Castellana, 261, 28046 Madrid, Spain; leticiagmzsnchz@gmail.com

**Keywords:** mediterranean diet, smartphone addiction, compulsive internet use, lifestyle factors, young adults

## Abstract

**Background/Objectives:** Problematic digital technology use may be associated with unhealthy dietary behaviors, but different forms of digital dependence may not relate to diet quality in the same way. This study examined the association between Mediterranean diet adherence and problematic smartphone, Internet, and video game use in young adults. **Methods:** A cross-sectional study was conducted in 496 adults aged 18–34 years participating in the EVA-Adic study. Mediterranean diet adherence was assessed using the 14-item Mediterranean Diet Adherence Screener (MEDAS). Problematic digital use was evaluated using the EDAS-18 for smartphone dependence, the Compulsive Internet Use Scale (CIUS), and the Questionnaire of Experiences Related to Video Games (CERV). Multivariable regression models were adjusted for sociodemographic, anthropometric, and lifestyle factors. **Results**: Of the participants, 339 had low-to-moderate Mediterranean diet adherence, and 157 had high adherence. In fully adjusted models, higher EDAS-18 scores were associated with lower MEDAS scores (β = −0.022; 95% CI: −0.041 to −0.003), whereas higher CIUS scores showed a small positive association with MEDAS scores (β = 0.025; 95% CI: 0.002 to 0.049). CERV scores and the global digital addiction index were not associated with overall Mediterranean diet adherence. At the individual dietary-component level, higher smartphone dependence was associated with lower compliance with fruit and sugar-sweetened beverage recommendations. **Conclusions:** In this cross-sectional sample of young adults, smartphone dependence and compulsive Internet use showed small associations with Mediterranean diet adherence in opposite directions, whereas problematic video-game use and the exploratory global composite were not associated with the overall MEDAS-14 score. The magnitude of the observed associations was modest; their clinical and public-health relevance remains uncertain, and the component-level findings should be considered exploratory in the context of multiple testing. These results do not establish causality and require confirmation in longitudinal, multicenter studies using objective and context-specific measures of digital use.

## 1. Introduction

The Mediterranean diet (MedDiet) is recognized as an optimal nutritional pattern and a comprehensive lifestyle model with metabolic and cardiovascular implications [1,2,3]. It is characterized by a high consumption of monounsaturated fats (mainly from extra virgin olive oil), fruits, vegetables, legumes, nuts and fish, along with a low intake of red meat and ultra-processed products. This dietary pattern has systemic antioxidant and anti-inflammatory effects [2,3]. A high adherence to the MedDiet, between 18 and 35 years of age, is a determining factor in mitigating premature cardiometabolic risk and consolidating healthy habits in the long term [4,5]. However, contemporary sociodemographic changes have led to a progressive move away from this traditional model, replacing it with dietary patterns of lower nutritional quality and an increase in sedentary behaviours [6,7].

At the same time, global digitalization has driven the emergence of screen-related behavioral addictions, constituting a priority challenge for public health and nutritional psychiatry [8,9]. The problematic and compulsive use of the internet, smartphones and video games has substantially reconfigured the management of free time and the behavior of the young population [10,11,12]. Biomedical studies over the past five years consistently suggest that greater exposure to screens is inversely associated with overall diet quality and, specifically, with lower adherence to the MedDiet [11,13]. However, the biological and behavioral mechanisms underlying this phenomenon are complex and multidimensional. A biological–behavioral axis has been proposed, whereby excessive technology use may alter eating patterns through the dysregulation of appetite-related hormones, such as leptin and ghrelin, thereby increasing hedonic hunger for energy-dense foods [14]. Likewise, the underlying impulsivity and deficit in inhibitory control characteristic of digital addictive behaviors undermine the ability to plan meals and make fresh food choices, inducing unstructured patterns [15].

At the epidemiological level, the evidence of this negative impact is substantial in young populations, so digital sedentary lifestyles displace the consumption of fruits and vegetables and significantly increase the intake of sweets and fast food [13,16]. In Mediterranean populations, the use of mobile devices and social networks directly during main meals has emerged as one of the distractions with the greatest predictive impact on the consumption of ultra-processed foods and the destructuring of the traditional diet [17,18]. Similarly, the specific profile of problematic use of video games (Gaming) is associated with prolonged sessions of immobility and automatic or unconscious eating (mindless eating), increasing the consumption of sugary drinks, easily accessible and simultaneously consumed with the game [19]. Despite the growing volume of publications, the translation of these findings to the population of young adults and university students has important scientific limitations. Evidence in this age group remains scattered, less homogeneous, and methodologically heterogeneous [20]. The vast majority of recent scientific studies use assessment tools such as the Mobile Phone Addiction Index, the Smartphone Addiction Scale or the Young Internet Addiction Test [8,20,21]. On the other hand, the applicability of specific instruments adapted to the Spanish-speaking population, such as the abbreviated version of the Smartphone Dependence and Addiction Scale (EDAS-18) [22] or the Questionnaire of Experiences Related to Video Games (CERV) [23], as well as using the Compulsive Internet Use Scale (CIUS) [24], their direct relationship with adherence to the MedDiet quantified by the 14-item standardized MEDAS questionnaire has been little explored [25]. This knowledge gap makes it impossible to establish whether the specific digital dependence profiles of young adults act in a coordinated way in the deterioration of the protective dietary pattern par excellence [25].

To overcome this limitation, the present study aims to evaluate the relationship between adherence to the Mediterranean diet (measured through the 14-item MEDAS questionnaire) and different dimensions of problematic screen use (smartphone dependence using EDAS-18, compulsive internet use using CIUS, and problematic experiences with video games using CERV) in young adults between 18 and 34 years of age. It is hypothesized that subjects with higher digital behavioral addiction scores will have lower adherence to the MedDiet, characterized by a maladaptive food consumption profile driven by the disruption of everyday technological habits.

## 2. Materials and Methods

### 2.1. Study Design

The results of this work are part of the EVA-Adic study. The protocol for the EVA-Adic study has been previously published [26]. The EVA-Adic study is a cross-sectional descriptive observational study carried out at the Primary Care Research Unit of Salamanca (APISAL). It is registered in ClinicalTrials.gov with the registration number NCT05866133 (registration date: 29 July 2023).

### 2.2. Study Population

**Figure 1:**
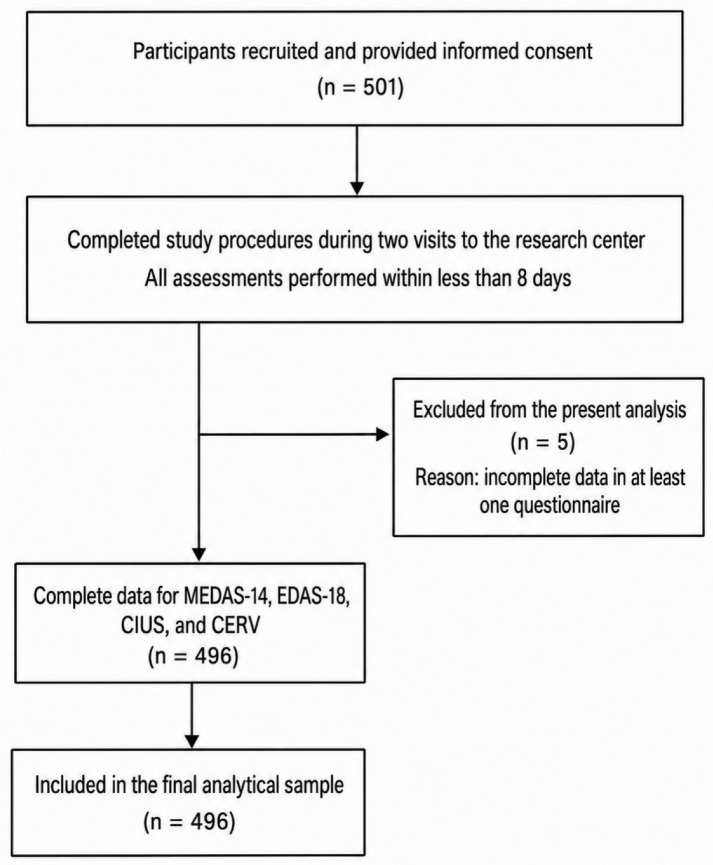
Flow diagram of participant inclusion in the present analysis.

Using consecutive sampling, 501 participants from the urban health area of Salamanca were recruited. Participants completed the study procedures during two visits to the research center, with all assessments performed within a period of less than eight days. Of the 501 participants recruited, 496 (99.0%) provided complete data for the MEDAS-14, EDAS-18, CIUS, and CERV questionnaires and were included in the final analytical sample. Five participants (1.0%) were excluded from the present analysis because of incomplete data in at least one of these instruments. Because of the cross-sectional design and the absence of a longitudinal follow-up period, no conventional losses to follow-up occurred. Inclusion criteria: age between 18 and 34 years of age and signing the informed consent form to participate in the study. Exclusion criteria: being in a terminal situation and not being able to travel to the research unit to carry out the tests. Figure 1. Flow diagram of participant inclusion in the present analysis. A total of 501 participants were recruited. Five participants were excluded because they had incomplete data for at least one of the four questionnaires evaluated. The final analytical sample comprised 496 participants.

### 2.3. Sample Size Calculation

*Sample Size Calculation.* Using version 7.2 of the GRANMO software and considering the MEDAS questionnaire score as the main outcome, accepting an alpha risk of 0.05 and a statistical power greater than 0.80 in a two-sided test, with 339 subjects in Group 1 (low adherence) and 157 in Group 2 (high adherence), the study was able to detect a difference equal to or greater than 0.54 units in the Mediterranean diet score. A common standard deviation of 2.01 was assumed for the Mediterranean diet score.

In carrying out this work, they have followed the Strengthening the Reporting of Observational Studies in Epidemiology (STROBE) [27] guidelines (the checklist is shown in Supplementary Table S1).

### 2.4. Variables and Measurement Instruments

The questionnaires used were collected from each participant in a period of less than 8 days. All tests were performed by 3 previously trained researchers in a standardized way, with the quality by an independent investigator. All evaluations were performed under standardized environmental conditions, and measurements were performed sequentially.

#### 2.4.1. Sociodemographic Variables and Personal History

Age and sex were recorded at the time of inclusion in the study using a structured clinical interview.

#### 2.4.2. Lifestyle Factors

Alcohol consumption was assessed using a structured, self-administered questionnaire that recorded the amount and type of alcohol commonly consumed during a week. The amount of alcohol consumed was measured in grams/week. Smoking was recorded following the standardized four-question questionnaire of the WHO MONICA study [28]. Physical activity was estimated with the International Physical Activity Questionnaire-Short Form (IPAQ-SF) [29]. It consists of seven questions about the type of physical activity performed during the last seven days and the total time spent on these activities. The result is the metabolic equivalent of the task per minute per week (MET-min/week).

#### 2.4.3. Mediterranean Diet

The MedDiet was assessed using the abbreviated Mediterranean Diet Adherence Screener (MEDAS) questionnaire of 14 items [25], validated and originally developed within the framework of the PREDIMED study (PREvención con DIeta MEDiterránea) by Martínez-González et al. [30], and extensively evaluated in the Spanish population [25]. This self-administered instrument is a validated nutritional screening tool that allows for a rapid and reproducible estimation of the degree of agreement of the participant’s eating habits with the traditional Mediterranean dietary pattern. The questionnaire consists of 14 closed questions that assess the frequency of consumption of essential foods and behaviors characteristic of the MedDiet pattern (Table S2 shows the 14 items evaluated). The overall score of the MEDAS is calculated by the arithmetic sum of all items, oscillating in a continuous range from 0 to 14 points, where a higher score reflects a greater qualitative and quantitative proximity to the Mediterranean standard. Following the standardized methodological criteria of the PREDIMED cohort [30,31], the participants’ adherence status was classified into two adherence categories: Low-to-moderate adherence: global score < 9 points; high adherence: global score ≥ 9 points.

#### 2.4.4. Screen Addiction and Dependence Assessment

To obtain a multidimensional assessment of the problematic use of technology and screens, three independently validated psychometric instruments were administered, and then the scores were combined to generate a Global Digital Addiction index, on a continuous scale.

*Smartphone Addiction: EDAS-18 Questionnaire.* Mobile phone dependence was determined using the validated Smartphone Dependence and Addiction Scale in its short version (EDAS-18) [22]. This instrument consists of 18 self-reported items that assess the dimensions of compulsive use, abstinence, tolerance, and interference with activities of daily living. Responses are recorded using a 5-point Likert scale (from 1 = “strongly disagree” to 5 = “strongly agree”). The continuous total score (edas18_total) is calculated by adding up all items, ranging from 18 to 90 points, where higher scores reflect a higher degree of smartphone addiction.

*Internet addiction: CIUS questionnaire.* Problematic Internet use in general was assessed through the *Compulsive Internet Use Scale* (CIUS) validated in European and Spanish settings [24]. The scale includes 14 items that examine loss of control, mental worry, the use of the internet as an emotional escape/regulation mechanism, and the resulting social or academic conflict. Each item is scored on a 5-point Likert scale (0 = “never” to 4 = “very often”). The continuous global score (cius_total) is obtained by adding the items, with a possible range from 0 to 56 points, denoting a greater severity of compulsive use the higher the score.

*Problematic video game use:* CERV questionnaire. Problematic experiences related to video game use were assessed using the Questionnaire of Experiences Related to Video Games (CERV) [23]. This self-administered instrument comprises 17 items assessing intrapersonal and interpersonal problems and difficulties related to excessive video game use. Each item is scored on a four-point Likert scale ranging from 0 (“never or almost never”) to 3 (“almost always”). The total score is calculated as the sum of the 17 items and ranges from 0 to 51 points, with higher scores indicating greater severity of problematic video game use.

*Calculation of the Global Digital Addiction Index.* The Global Digital Addiction Score was calculated as the sum of the raw EDAS-18, CIUS, and CERV total scores.

Internal consistency of the instruments was assessed in the present sample using Cronbach’s alpha. Complete item-level data were available for 496 participants for the MEDAS-14, 496 for the EDAS-18, 496 for the CIUS, and 496 for the CERV. Cronbach’s alpha coefficients were 0.437 for the MEDAS-14, 0.886 for the EDAS-18, 0.927 for the CIUS, and 0.893 for the CERV. For the MEDAS-14, which comprises dichotomous items, Cronbach’s alpha is equivalent to the Kuder–Richardson 20 coefficient. The comparatively lower coefficient observed for the MEDAS-14 should be interpreted in light of its nature as a composite dietary adherence index comprising heterogeneous dietary behaviors rather than a strictly unidimensional psychometric scale.

### 2.5. Statistical Analysis

*Statistical analyses* were performed using IBM SPSS Statistics software for Windows, version 30.0 (IBM Corp., Armonk, NY, USA). The threshold of statistical significance was set at *p* < 0.05 for all two-sided tests. Quantitative variables were expressed as means and standard deviations (SD), while categorical and nominal variables were summarized using absolute frequencies (*n*) and percentages (%). To explore possible variations in baseline sample characteristics, comparative analyses were carried out by stratifying participants according to their adherence to the MedDiet (Adherents vs. Non-adherent) and according to sex. Differences between groups for categorical variables were assessed using the Chi-square test. For continuous variables, Student’s *t*-tests were applied for independent samples. The dependent variable (global MedDiet adherence score, MEDAS) and all digital addiction metrics (EDAS-18, CIUS, CERV, and the global digital addiction index) were treated as continuous variables to preserve the total statistical power and inherent variability of the psychometric and nutritional scales, thus avoiding the arbitrary loss of information associated with categorization. Initially, bivariate associations between the global score of the Mediterranean diet and the four digital addiction scales were assessed using Pearson’s correlation coefficient (r). Subsequently, multivariable linear regression models were constructed to explore the independent predictive value of digital addictions on adherence to the Mediterranean diet. To assess potential confounding effects, a hierarchical regression approach was employed through two sequential adjustment levels: Model 1 was adjusted for major sociodemographic factors (age and sex); Model 2 was additionally adjusted for anthropometric and lifestyle covariates, including alcohol consumption (grams/week), physical activity levels assessed by the International Physical Activity Questionnaire (IPAQ), smoking status, and Body Mass Index (BMI). To ensure the absence of multicollinearity between predictors and covariates within the fully adjusted equations, the Variance Inflation Factor (VIF) was explicitly calculated, considering a VIF value < 2.0 as the threshold for accepting a correspondingly low collinearity. To assess whether sex acted as a moderating variable in the relationship between measures of digital addiction and adherence to the Mediterranean Diet, multiplicative interaction terms (sex × digital addiction score) were explicitly calculated for ex × digital addiction score the multivariable linear models analyzed. These terms were introduced into a final hierarchical block in multiple regressions. In case of detecting a statistically significant interaction (*p* < 0.05), we planned to perform independent sex-stratified analyses to decipher specific patterns. Otherwise, the global joint models duly adjusted for this covariate were reported. Finally, to unravel exploratory component-level associations, independent multivariate binary logistic regression analyses were performed for each of the 14 individual dietary components that make up the MEDAS questionnaire (treated as dichotomous outcome variables: 0 = non-compliance; 1 = compliance with the healthy criterion). These item-by-item regressions assessed the specific impact of each digital addiction metric using the covariate fit structure detailed in Model 2. The results of these logistic equations were expressed as *Odds Ratios (ORs)* together with their corresponding 95% Confidence Intervals (95% CI).

### 2.6. Ethical Considerations

The Ethics Committee in Drug Research of the Salamanca Health Area approved this project on 10 July 2021 (CEIm Reference Code Ref. PI 2021 088671048) and on 24 July 2023 (CEIm Reference Code Ref. PI 2023 071332). Throughout the study, the standards of good practice in observational studies established by the Declaration of Helsinki and by the WHO were carried out [32]. The confidentiality of the participants was always guaranteed in accordance with Organic Law 3/2018, European Regulation 2016/679 and Council Directive 27 April 2016 on Data Protection. All the subjects analyzed signed informed consent before being included in the study and after receiving detailed information about the procedures that would be performed.

### 2.7. Use of Artificial Intelligence Tools

During the preparation of the manuscript, ChatGPT (OpenAI, version 5.5) was used to help with the English edition. Generative AI was not used to create scientific images, generate data, perform analysis, or alter study results. All results and statistical analyses were performed by the authors. The authors reviewed and approved all AI-assisted edits and assume full responsibility for the final manuscript.

## 3. Results

Table 1 shows the sociodemographic, anthropometric and behavioural characteristics of the sample according to the level of adherence to the Mediterranean Diet. Of the 496 participants included, 339 had low adherence to the Mediterranean pattern, and 157 had high adherence. Participants with high adherence were older and had a higher proportion of women, a lower prevalence of smoking and a lower BMI compared to the low-adherence group. No differences were observed between the groups in alcohol consumption or physical activity levels. Regarding the digital addiction scales, the EDAS-18, CIUS, CERV scores and the global digital addiction score were similar between the groups.

Table 2 presents the characteristics of the participants according to sex. Men were older and showed higher values of body mass index, alcohol consumption and physical activity. No differences were observed in smoking. Regarding the variables related to the problematic use of technologies, no differences were detected by sex in EDAS-18, CIUS or in the global problematic use of screens. However, men had higher scores in CERV, indicating a greater problematic use of video games.

**Figure 2:**
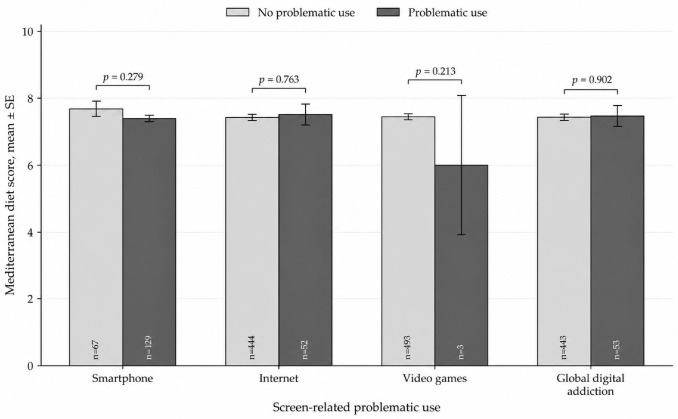
Mediterranean diet adherence score according to problematic screen use. Bars represent the mean Mediterranean diet score ± standard error according to the presence or absence of problematic use of smartphones, internet, video games, and global digital addiction. Comparisons between groups were performed using Student’s *t*-test. MEDAS: Mediterranean Diet Adherence Screener; SE: standard error.

Figure 2 shows the differences in the Mediterranean Diet adherence score according to the presence or absence of problematic screen use. No differences in MEDAS score were observed between participants with and without problematic use of smartphones, the internet, video games or global digital addiction.

**Figure 3:**
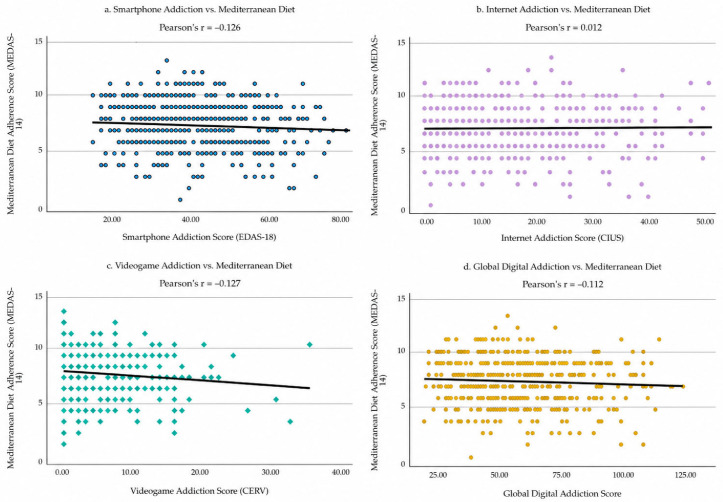
Correlations between digital addiction-related scores and Mediterranean diet adherence. Scatter plots showing the association between MEDAS-14 score and: (**a**) smartphone addiction assessed using EDAS-18; (**b**) Internet addiction assessed using CIUS; (**c**) videogame addiction assessed using CERV; and (**d**) global digital addiction score. The black line represents the linear regression trend. Pearson’s correlation coefficients are shown for each panel. MEDAS-14: 14-item Mediterranean Diet Adherence Screener; EDAS-18: Smartphone Addiction Scale; CIUS: Compulsive Internet Use Scale; CERV: Questionnaire of Experiences Related to Video Games.

Figure 3 shows the correlation between adherence to the Mediterranean diet, assessed using the MEDAS-14 questionnaire, and digital addiction scores. A weak inverse correlation was observed between the MEDAS-14 score and smartphone addiction measured by EDAS-18 (*r* = −0.126; *p* = 0.005), video game addiction assessed by CERV (*r* = −0.127; *p* = 0.005), and the global digital addiction score showed a weak negative correlation with adherence to the Mediterranean diet (*r* = −0.112; *p* = 0.013). In contrast, Internet addiction assessed by CIUS showed a positive correlation with MEDAS-14 (*r* = 0.012; *p* = 0.790).

**Figure 4:**
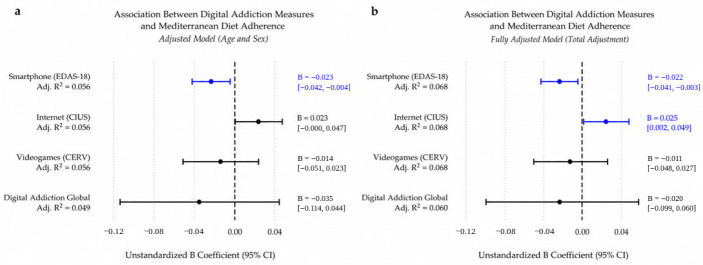
Association between measures of digital addiction and adherence to the Mediterranean diet. The non-standardized β coefficients and their 95% confidence intervals are shown for each measure of digital addiction. Panel (**a**) shows the model adjusted for age and sex. Panel (**b**) shows the model with full adjustment. EDAS-18: smartphone addiction scale; CIUS: Compulsive Internet Use Scale; CERV: questionnaire of experiences related to video games; CI: confidence interval; Adjusted R^2^: adjusted coefficient of determination. Estimates in blue indicate statistically significant associations.

As shown in Figure 4, smartphone addiction was inversely associated with the MEDAS questionnaire score in both the age- and sex-adjusted model and the fully adjusted model. In separate fully adjusted models, higher EDAS-18 scores were associated with slightly lower MEDAS-14 scores, whereas higher CIUS scores were associated with slightly higher MEDAS-14 scores. CERV scores and the exploratory global raw-score composite were not associated with overall Mediterranean diet adherence. The magnitude of both statistically significant associations was small: a 10-point difference in EDAS-18 corresponded to an approximately 0.22-point difference in MEDAS-14, and a 10-point difference in CIUS corresponded to a 0.25-point difference.

In order to determine whether the impact of problematic use of technology on dietary patterns differed between men and women, the effects of interaction with sex were evaluated for each psychometric scale individually and for the global construct. In no case did the terms of interaction reach statistical significance (Model 1 [Smartphone]: *p* = 0.668; Model 2 [Internet]: *p* = 0.361; Model 3 [Video games]: *p* = 0.790; Model 4 [Global]: *p* = 0.578). None of the sex-by-digital addiction interaction terms reached statistical significance. Therefore, no statistical evidence of effect modification by sex was observed in the present sample. However, non-significant interaction tests should not be interpreted as evidence that the associations are equivalent in men and women, particularly because the study may have had limited statistical power to detect small interaction effects. Accordingly, the overall models adjusted for sex are presented, while potential sex-specific differences should be further examined in larger studies specifically powered for interaction analyses.

**Figure 5:**
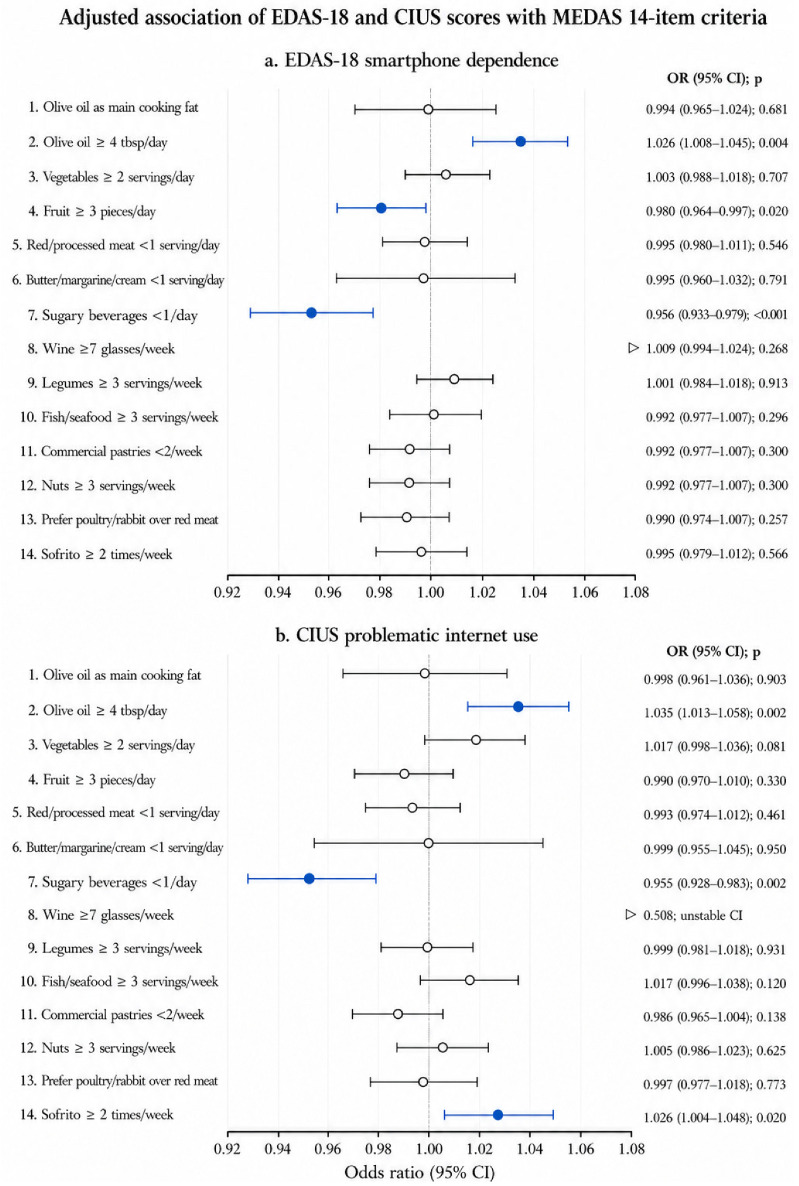
Adjusted association of EDAS-18 and CIUS scores with individual 14-item MEDAS criteria. The odds ratio (*OR*) and 95% confidence intervals for the fulfillment of each MEDAS criterion are shown for each one-point increase in the EDAS-18 or CIUS score. Panel (**a**) represents associations with smartphone dependence as measured by EDAS-18, and panel (**b**) represents associations with problematic internet use as measured by CIUS. Model 2 was adjusted for age, sex, body mass index, smoking, alcohol consumption, and physical activity. Blue circles and lines indicate statistically significant associations; Gray circles and lines indicate non-significant associations. The wine consumption criterion had unstable confidence intervals and is shown only as an indication.

**Figure 6:**
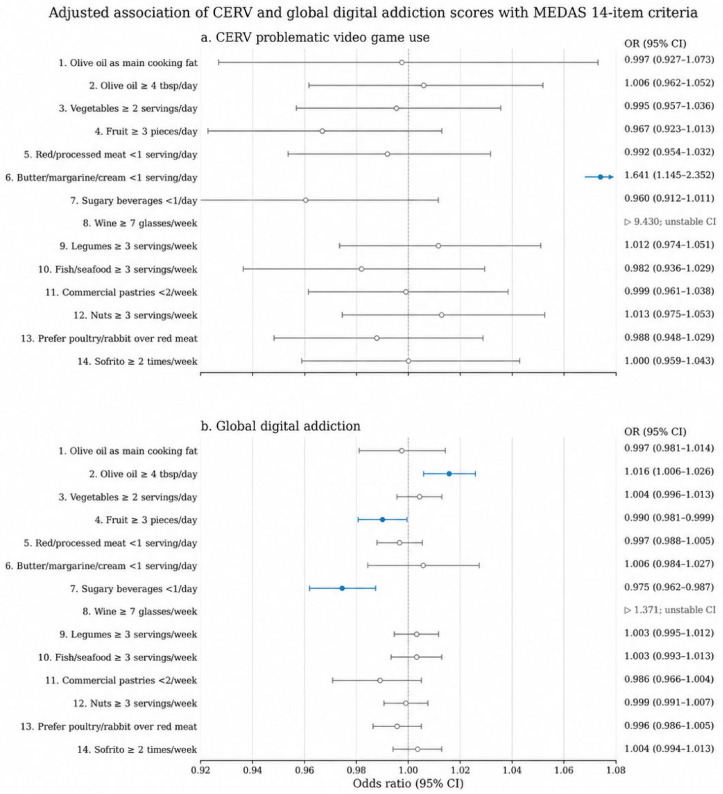
Adjusted association of CERV and global digital addiction scores with individual MEDAS 14-item criteria. Odds ratios (*ORs*) and 95% confidence intervals (95% CIs) are shown for the association between digital addiction scores and each Mediterranean Diet Adherence Screener (MEDAS-14) criterion. Panel (**a**) shows the association for problematic videogame use measured by the CERV score, and panel (**b**) shows the association for the global digital addiction score. Blue estimates indicate statistically significant associations, whereas grey estimates indicate non-significant associations. For the wine criterion, the confidence interval was unstable and is therefore indicated separately. CERV: Video Game Experiences Questionnaire; MEDAS: Mediterranean Diet Adherence Screener; OR: odds ratio; CI: confidence interval.

The component analyses presented in Figure 5 and Figure 6 were exploratory, and the *p* values are nominal.

Figure 5 shows the adjusted association between EDAS-18 and CIUS scores and compliance with each of the 14 criteria of the MEDAS questionnaire using the total fit model. A higher score on EDAS-18 was associated with a higher probability of meeting the criterion for olive oil consumption ≥ 4 tablespoons/day *(OR = 1.026). Conversely, higher EDAS-18 scores were associated with a lower probability of meeting the fruit consumption criterion ≥3 pieces/day (OR = 0.980)* and the criterion of low consumption of sugary drinks (<1/day) (*OR = 0.956*). No significant associations were observed with the rest of the MEDAS criteria. Similarly, a higher CIUS score was associated with a higher probability of meeting the criterion for olive oil consumption ≥ 4 tablespoons/day (*OR = 1.035*) and sofrito consumption ≥ 2 times/week (*OR = 1.026*). However, an inverse association was also observed with the criterion of low consumption of sugar-sweetened beverages (<1/day) (*OR = 0.955*). The criterion related to wine consumption had unstable confidence intervals and should therefore be interpreted with caution and was not considered conclusive.

Figure 6 shows the adjusted association between CERV and global digital addiction scores with compliance with each of the 14 criteria of the MEDAS questionnaire using the total fit model. A higher score on CERV was associated with a higher probability of meeting the criterion of low consumption of butter, margarine or cream (<1 serving/day) (*OR = 1.641*). No significant associations were observed between CERV and the rest of the MEDAS criteria. The criterion for wine consumption had unstable confidence intervals and should therefore be interpreted with caution. In relation to global digital addiction, higher scores were significantly associated with a higher probability of meeting the criterion for olive oil consumption ≥ 4 tablespoons/day (*OR = 1.016*). Conversely, a higher global digital addiction score was associated with a lower probability of meeting the fruit consumption criterion ≥ 3 pieces/day (*OR = 0.990*) and the criterion of low consumption of sugary drinks (<1/day) (*OR = 0.975*). No associations were observed with the other components of MEDAS.

However, exploratory item-level analyses identified several nominally significant associations with individual MEDAS criteria. However, because multiple comparisons were performed and no multiplicity correction was applied, these results should be interpreted as hypothesis-generating and not as evidence that specific dietary components mediate or explain the overall associations.

## 4. Discussion

The principal finding is that different digital-use constructs showed small associations with Mediterranean diet adherence in different directions. The inverse EDAS-18 association was directionally consistent with a less favorable dietary pattern, but its magnitude was modest. The positive CIUS association was unexpected, small, and may reflect residual confounding, measurement differences, or chance. Therefore, neither association should be considered clinically meaningful without confirmation in longitudinal studies. From a conceptual perspective, smartphone use during meals may influence eating behavior through attentional distraction and context-specific patterns of use. Experimental evidence has shown that using a smartphone while eating can increase energy and fat intake, while ecological and population-based studies indicate that its dietary effects may vary according to the specific function, duration, and content of smartphone use [17,33,34]. These device- and context-specific mechanisms may partly explain why smartphone dependence showed a less favorable association with diet quality in our sample than compulsive Internet use assessed as a broader construct. The positive association observed with CIUS should be interpreted with caution: it does not necessarily imply a healthy effect of problematic internet use, but may reflect differences in the type of online activity, in the profile of the participants, in access to nutritional information or in residual factors not fully captured by the models. Overall, EVA-Adic provides a relevant message for behavioral nutrition research: it is not enough to measure “screen time” or “digital addiction” as unique constructs; It is necessary to differentiate between the device, content, purpose of use, time of day and food context.

The inverse association between EDAS-18 and adherence to the MetDiet is consistent with recent literature that relates greater exposure to screens with poorer diet quality. In the Spanish population, the PASOS study showed that more screen time was associated with poorer adherence to the MetDiet, including lower consumption of foods characteristic of the Mediterranean pattern such as fruits, vegetables, fish, legumes and nuts [35]. Along the same lines, a study published in Italy found that certain digital activities, especially digital games and time spent on television or streaming platforms, were associated with lower adherence to the Mediterranean diet [16]. In addition, several publications indicate that the problematic use of mobile devices alters eating routines and is inversely associated with diet quality [8,11]. The specific association found in this study between EDAS-18 scores, reduced fruit consumption, and increased sugar-sweetened beverages supports the biological-behavioral axis theory proposed by Helvacı and Tayhan (2025) [13]. According to this model, the constant distraction of screens and the use of mobile devices directly during main meals encourage unstructured and unconscious eating (mindless eating), displacing fresh foods that require preparation for ultra-processed options that are easy to consume simultaneously [17,18]. On the other hand, although EVA-Adic focuses on addictive behavior scales and not only on exposure time, these results support a common interpretation: the digitalization of daily habits can displace protective eating routines and favor less structured eating behaviors. There are also prospective data that reinforce this hypothesis. In a large cohort of U.S. adolescents, different screen time modalities, including television, video games, videos, messages, and social media, were associated cross-sectionally and prospectively with lower overall diet quality as measured by a MIND index, which shares elements with the Mediterranean pattern and with the DASH dietary approaches to stop hypertension (DASH) diet [36]. Likewise, in Turkish adolescents, it has been described that levels of digital addiction tend to decrease as adherence to the Mediterranean diet and physical activity increase, suggesting that digital addiction is usually integrated into a cluster of lower behavioral health: more sedentary lifestyle, greater probability of eating during the use of social networks, more nighttime intake and less adherence to healthy dietary patterns [13]. These findings are consistent with the negative direction observed between EDAS-18 and MEDAS-14 in EVA-Adic. The literature on smartphones and eating behavior offers plausible mechanisms to explain this association. A recent systematic review found that problematic smartphone use and longer daily use time are associated with more symptoms of eating disorders, body dissatisfaction, emotional eating, loss of control, and food addiction, although the overall certainty of the evidence is limited by the predominance of cross-sectional designs [37]. Observational studies in university students have also described relationships between smartphone addiction, risk of eating disorders, poorer sleep quality, evening chronotype and lifestyle changes [21,38]. In this context, the smartphone can act as a marker of impulsivity, emotional dysregulation, insufficient sleep, exposure to food marketing, social comparison and distracted eating, all factors capable of deteriorating adherence to a structured dietary pattern.

The main discrepancy of EVA-Adic with respect to part of the literature is the positive association between CIUS and MEDAS-14. In adolescents, a study of mediation and network analysis observed that problematic use of the internet was associated with lower adherence to the MetDiet and that diet quality partially mediated the relationship between problematic use of the internet and risk of altered eating behavior [39]. Similarly, Helvacı and Tayhan’s work found an inverse relationship between digital addiction and adherence to the MetDiet [13]. At first glance, these data contrast with the positive result observed for CIUS in EVA-Adic. However, there are several reasons that may explain this apparent discrepancy. First, CIUS measures compulsive use of the internet in a broad sense, but does not distinguish whether the use is oriented towards social networks, search for information, work, study, social interaction, shopping, passive leisure or health content. It is possible that, in certain profiles, greater use of the internet coexists with greater exposure to information on nutrition, healthy recipes, physical activity or Mediterranean eating patterns. Second, the scales used in other studies are not necessarily equivalent to CIUS, and much research has been carried out in adolescents or university students, so extrapolation to the EVA-Adic sample should be prudent. Third, some recent studies have not found a direct relationship between Mediterranean adherence and internet addiction. For example, a pilot study published in Nutrients in Greek adolescents and university students observed that Mediterranean adherence was not significantly associated with internet addiction or altered eating behavior; On the other hand, female sex and moderate internet addiction were associated with an increased risk of disordered eating [11]. This result supports the idea that the relationship between the internet, the Mediterranean diet and eating behavior is complex and may depend on the digital phenotype evaluated. For all these reasons, the positive association observed between CIUS and MEDAS-14 should be considered exploratory and interpreted with caution. CIUS assesses compulsive Internet use as a broad construct but does not characterize the content accessed, the purpose of use, the device employed, or the context in which Internet use occurs [24]. Young adults commonly use social media, websites, and Internet search engines to access nutrition information, recipes, and health-related content; however, exposure, intentionality, engagement, and the quality of such content vary substantially [40]. Therefore, although greater exposure to nutrition-related information, healthy recipes, physical activity content, or Mediterranean dietary recommendations could hypothetically contribute to this association in some participants, this explanation cannot be confirmed with the data collected. Moreover, the magnitude of the association was small, and residual confounding or chance may also have contributed. Consequently, the result should not be interpreted as evidence that compulsive Internet use has a beneficial effect on diet quality. Future studies should characterize digital activity according to content, purpose, device, timing, intentionality, engagement, and eating context to clarify this unexpected association. This cautious interpretation is particularly relevant for the positive association between CIUS and MEDAS-14, which was small and unexpected and should be considered exploratory rather than evidence of a beneficial effect of compulsive Internet use.

On the other hand, the lack of significant association of video game score (CERV) with global diet differs from that reported by Ribeiro et al. [19], who associate the gamer’s profile with a high consumption of energy drinks and fast foods. In our sample, although men scored significantly higher on the CERV, problematic video game use was only associated in isolation with low consumption of butters or cream, suggesting that in a general college population (not exclusively esports) the pattern of problematic gaming might be more segregated from the household’s core shopping and eating habits. These opposing associations underscore that not all digital users share the same nutritional risk. Therefore, the results of EVA-Adic invite us to abandon a simplistic approach based exclusively on “more digital use = worse diet” and to move towards more precise digital phenotypes: compulsive smartphone, academic/work internet, social networks, streaming, gaming, night use, use during meals and exposure to food content.

Nor can we forget that psychological and sleep-related factors may also contribute to the observed associations. Anxiety, depressive symptoms, perceived stress, and poor sleep quality have been associated with problematic smartphone and Internet use in young adults [41,42]. These factors have also been linked to less favorable dietary behaviors, including emotional eating and poorer adherence to healthy dietary patterns, whereas greater adherence to the Mediterranean diet has been associated with better sleep quality and lower levels of depression, anxiety, and stress [43]. Because these variables were not assessed in the present analysis, residual confounding cannot be excluded. It is also possible that psychological distress or sleep disturbances partly mediate the relationship between problematic digital use and adherence to the Mediterranean diet, although the cross-sectional design does not allow these pathways to be established. Future studies should include validated measures of mental health, sleep quality, and chronotype and should explore potential mediation and moderation pathways.

Although several associations reached statistical significance, their magnitude was generally small. A 10-point increase in EDAS-18 was associated with an estimated 0.22-point decrease in the MEDAS-14 score, whereas a 10-point increase in CIUS was associated with an estimated 0.25-point increase. These differences are modest relative to the full 0–14 range of the MEDAS-14. Similarly, most statistically significant odds ratios for the individual MEDAS components were close to 1.00, indicating small changes in the odds of meeting each dietary criterion. Therefore, statistical significance should not be equated with clinical or public health relevance, which remains uncertain. The findings should be interpreted as preliminary evidence of differential behavioral patterns rather than as demonstrating substantial effects of problematic digital use on diet quality.

*Clinical and public health implications.* These findings may support the inclusion of context-specific digital-use measures in future nutritional research. However, the small effect sizes and cross-sectional design do not currently justify specific clinical screening or intervention recommendations based on these results alone. From a clinical point of view, the results suggest that “screen time” or “digital addiction” should not be evaluated as a homogeneous block, since each device and technological behavior modulates health habits in different ways. The negative association between EDAS-18 and MEDAS-14 may suggest that the smartphone could act as a warning sign of lower adherence to healthy eating habits. In consultation, this can be translated into simple and applicable recommendations: telephone-free meals, structured digital schedules, reduction in nighttime use, conscious eating strategies, planning of Mediterranean menus and replacing ultra-processed snacks with options in line with the Mediterranean pattern [14,15]. However, the positive association with CIUS makes it necessary to avoid excessively reductionist clinical messages. The Internet can also be a tool for health promotion if it is channeled into nutritional education, goal tracking, healthy recipes, support communities, telemonitoring or brief digital interventions. Recent studies have indicated that smartphone addiction can be inversely related to health-promoting behaviors and worse intuitive eating [44]. Consequently, the clinical message should not only be to “reduce screens”, but to “order digital use”, separating disruptive, impulsive and nocturnal use from informative, planned and self-care oriented use. *From a public health perspective*, these findings suggest that the promotion of the Mediterranean diet may benefit from interventions that integrate digital literacy, food literacy, and online food marketing education. Mediterranean pattern is associated with better quality of life in adults in recent reviews published in Nutrients [45], and a healthy diet is associated with better sleep quality, while processed foods rich in free sugars are associated with worse sleep characteristics [46]. Since sleep, physical activity, digital use and diet form an interdependent behavioral system, the most promising interventions are likely to be multicomponent: limiting smartphone use at critical moments, improving sleep hygiene, increasing physical activity and reinforcing Mediterranean habits.

*Limitations of the study.* This study has several limitations that must be recognized. First, the cross-sectional design prevents causality or directionality from being established. It is not possible to determine whether problematic smartphone use leads to less Mediterranean adherence, whether poorer organization of habits favors greater digital vulnerability, or whether both phenomena share common determinants such as stress, anxiety, insufficient sleep, sedentary lifestyle, or emotional dysregulation. Second, the primary variables are based on self-reported questionnaires, which can introduce recall bias, social desirability, and misclassification. Although MEDAS-14 is a useful and brief tool, it does not replace more detailed dietary methods or allow you to fully capture total energy, portion sizes, ultra-processed foods, hourly eating patterns or episodes of distracted eating. Third, digital addiction scales assess related but not identical dimensions. EDAS-18, CIUS and scales linked to video games or gaming are not interchangeable, so the associations should be interpreted as specific to each construct. Fourth, objective measures of screen time, mobile unlocks, night-time use, specific applications, exposure to food content or use during meals are not necessarily available. This absence limits the identification of mechanisms. Fifth, although adjusted models reduce confusion, residual confounding by socioeconomic level, weight status, anxiety, depression, perceived stress, sleep quality, physical activity, shifts, coexistence, educational level or motivation towards health cannot be ruled out. Also, the consecutive recruitment of participants from a single urban area may limit the external validity of the study. Therefore, the findings should be generalized with caution to populations from other geographic, rural, socioeconomic, or sociocultural contexts. Multicenter studies with more diverse samples are warranted.

Finally, the sample size of 496 subjects is adequate for detecting global associations, but certain stratified analyses or interactions may require larger samples to avoid unstable estimates. *In summary*, the results of EVA-Adic provide novel evidence by showing that digital addictive behaviors are differentially related to adherence to the Mediterranean diet. The inverse association between EDAS-18 and MEDAS-14 is consistent with recent studies linking intensive or problematic screen use to poorer diet quality. On the other hand, the positive association between CIUS and MEDAS-14 introduces a relevant nuance: problematic use of the internet should not automatically be assumed as equivalent to problematic use of smartphones. These findings support the need to analyse the different digital phenotypes separately and to incorporate digital behaviour into the promotion strategies of the Mediterranean diet. Future longitudinal studies, with objective measures of digital use and more detailed dietary assessment, will clarify the causal direction and design personalized interventions that integrate digital health and preventive nutrition.

## 5. Conclusions

In this cross-sectional sample of young adults, smartphone dependence and compulsive Internet use showed small associations with Mediterranean diet adherence in opposite directions, whereas problematic video-game use and the exploratory global composite were not associated with the overall MEDAS-14 score. The magnitude of the observed associations was modest; their clinical and public-health relevance remains uncertain, and the component-level findings should be considered exploratory in the context of multiple testing. These results do not establish causality and require confirmation in longitudinal, multicenter studies using objective and context-specific measures of digital use.

## Figures and Tables

**Table 1:** Sociodemographic, anthropometric, behavioral, and digital addiction characteristics of the sample according to Mediterranean diet adherence level.

Variables and Instruments	Total Sample (N = 496)	Low Adherence (MEDAS < 9; *n* = 339)	High Adherence (MEDAS ≥ 9; *n* = 157)	*p*-Value ^a^
Age (years), mean ± SD	26.60 ± 4.41	26.29 ± 4.53	27.28 ± 4.06	0.015 *
Sex, *n* (%)				<0.001 *
Male	220 (44.4)	171 (50.4)	49 (31.2)	
Female	276 (55.6)	168 (49.6)	108 (68.8)	
Alcohol consumption (g/week), mean ± SD	48.93 ± 73.90	52.19 ± 74.03	41.88 ± 73.36	0.149
Physical activity (MET-min/week), mean ± SD	2660.50 ± 2499.48	2623.46 ± 2739.65	2740.49 ± 1884.60	0.628
Smoking status, *n* (%)				0.002 *
Non-smoker	407 (82.1)	266 (78.5)	141 (89.8)	
Smoker	89 (17.9)	73 (21.5)	16 (10.2)	
BMI (kg/m^2^), mean ± SD	24.01 ± 3.80	24.30 ± 3.96	23.38 ± 3.35	0.008 *
Digital addiction scales				
EDAS-18 (smartphone), mean ± SD	40.87 ± 12.24	41.02 ± 12.63	40.57 ± 11.37	0.703
CIUS (Internet), mean ± SD	13.83 ± 9.89	13.57 ± 9.50	14.38 ± 10.70	0.418
CERV (video games), mean ± SD ^b^	3.54 ± 5.22	3.80 ± 5.29	2.98 ± 5.06	0.103
Global digital addiction score, mean ± SD	58.25 ± 21.76	58.39 ± 21.77	57.93 ± 21.80	0.826

Values are presented as mean ± standard deviation or *n* (%). Abbreviations: SD, standard deviation; BMI, body mass index; MEDAS, Mediterranean Diet Adherence Screener; EDAS-18, Smartphone Addiction Scale; CIUS, Compulsive Internet Use Scale; CERV, Video Game Experiences Questionnaire. ^a^ *p*-values were calculated using the χ^2^ test for categorical variables and independent-samples *t*-tests for continuous variables, with Welch’s correction when variances were unequal. ^b^ CERV scores were available for the full analytic sample (N = 496). * Statistically significant difference (*p* < 0.05).

**Table 2:** Sociodemographic, anthropometric, behavioral, and digital addiction characteristics of the sample according to sex.

Variables and Instruments	Men (*n* = 220)	Women (*n* = 276)	*p*-Value ^a^
Age (years), mean ± SD	27.09 ± 4.40	26.22 ± 4.38	0.029 *
Score Mediterranean diet, mean ± SD	6.96 ± 2.06	7.81 ± 1.88	<0.001 *
Alcohol consumption (g/week), mean ± SD	61.56 ± 91.54	38.86 ± 54.11	0.001 *
Physical activity (MET-min/week), mean ± SD	3172 ± 3140	2253 ± 1737	<0.001 *
Smoking status, *n* (%)			0.413
Non-smoker	184 (83.6)	223 (80.8)	
Smoker	36 (16.4)	53 (19.2)	
BMI (kg/m^2^), mean ± SD	25.30 ± 3.67	22.98 ± 3.59	<0.001 *
Digital Addiction Scales			
EDAS-18 (smartphone)	40.37 ± 12.15	41.28 ± 12.31	0.411
CIUS (internet)	13.38 ± 9.75	14.18 ± 10.01	0.370
CERV (video games)	5.94 ± 6.18	1.63 ± 3.23	<0.001 *
Global digital addiction	59.69 ± 22.09	57.09 ± 21.46	0.187

Values are presented as mean ± standard deviation or *n* (%). SD: standard deviation; BMI: body mass index; MET: metabolic equivalent of task; EDAS-18: Smartphone Dependency and Addiction Scale; CIUS: Compulsive Internet Use Scale; CERV: Video Game Experiences Questionnaire. ^a^ *p*-values were calculated using Student’s *t*-test for continuous variables, with Welch’s correction when variances were unequal, and the chi-square test for categorical variables. * *p* < 0.05.

## Data Availability

The data supporting the findings of this study are available on ZENODO under the https://doi.org/10.5281/zenodo.16910594, https://zenodo.org/records/16910594 (accessed on 23 May 2026).

## Supplementary Materials

The following supporting information can be downloaded at https://www.mdpi.com/article/10.3390/nu18152425/s1, Table S1: STROBE checklist for the EVA-Adic cross-sectional study and Table S2: Criteria for Efficiency and Scoring of the 14-item Mediterranean Diet Adherence Screen (MEDAS).

## Abbreviations

APISAL	Primary Care Research Unit of Salamanca
BMI	Body Mass Index
CERV	Video Game Experiences Questionnaire
CIUS	Compulsive Internet Use Scale
EDAS-18	Smartphone Dependency and Addiction Scale, short 18-item version
IBSAL	Institute of Biomedical Research of Salamanca
IPAQ-SF/IPAQ	International Physical Activity Questionnaire–Short Form
MEDAS/MEDAS-14	Mediterranean Diet Adherence Screener
MedDiet	Mediterranean Diet
METs/MET	Metabolic Equivalent of Task
OR	Odds Ratio
PREDIMED	Prevention with Mediterranean Diet
RICAPPS	Network for Research on Chronicity, Primary Care, and Health Promotion
SACyL	Regional Health Management of Castile and León
SD	Standard Deviation
SE	Standard Error
STROBE	Strengthening the Reporting of Observational Studies in Epidemiology
VIF	Variance Inflation Factor
WHO	World Health Organization

## Appendix A

EVA-Adic Investigators Group: The members of the EVA-Adic Group are: Manuel A. Gómez Marcos, Luis García-Ortiz, Emiliano Rodríguez-Sánchez, Cristina Lugones-Sánchez, Olaya Tamayo-Morales, Susana González-Sánchez, Leticia Gómez-Sánchez, Sara M. Vicente-Gabriel, Alberto Vicente-Prieto, Sandra Conde-Martín, Marta Gómez-Sánchez, Elena Navarro Matias, Carmen Patino-Alonso, José A. Maderuelo-Fernández, Angela de Cabo-Laso, Benigna Sanchez-Salgado, and Laura Fernandez-Matas.
