# Supplementary material for: Smartphone and Internet Addictive Behaviors Are Differentially Associated with Mediterranean Diet Adherence in Young Adults: The EVA-Adic Study
Source: Nutrients. 2026 Jul 24;18(15):2425. doi: 10.3390/nu18152425 (PMC13467619; doi:10.3390/nu18152425)
Supplement: Supplementary file 1 [file nutrients-18-02425-s001.zip › nutrients-4428831-supplementary.pdf]

Supplementary Table S1. STROBE checklist for the EVA-Adic cross-sectional study

Section/topic	Item	STROBE recommendation	Reported in manuscript	Comments for this manuscript
Title and abstract	1a	Indicate the study design with a commonly used term in the title or the abstract.	Title and Abstract	The title identifies the study context (EVA-Adic Study) and the abstract describes the study as an observational, descriptive, cross-sectional study.
Title and abstract	1b	Provide in the abstract an informative and balanced summary of what was done and what was found.	Abstract	The abstract includes background/objectives, methods, sample, principal findings and conclusions. However, the conclusions currently contain Spanish text and should be fully translated before submission.
Introduction	2	Explain the scientific background and rationale for the investigation being reported.	Introduction	The Introduction describes the Mediterranean diet, screen-related behavioral addictions, plausible behavioral mechanisms, and the gap regarding digital dependence profiles and MedDiet adherence.
Introduction	3	State specific objectives, including any prespecified hypotheses.	End of Introduction	The objective is to evaluate the relationship between MEDAS-14 adherence and smartphone dependence, compulsive internet use and videogame-related problems in young adults. A hypothesis of lower MedDiet adherence with higher digital addiction scores is stated.

Section/topic	Item	STROBE recommendation	Reported in manuscript	Comments for this manuscript
Methods	4	Present key elements of study design early in the paper.	Methods, 2.1 Study Design	The EVA-Adic study is described as a cross-sectional descriptive observational study conducted at APISAL and registered in ClinicalTrials.gov (NCT05819840).
Methods	5	Describe the setting, locations and relevant dates, including periods of recruitment, exposure, follow-up and data collection.	Methods, 2.1-2.2	The study setting is the Primary Care Research Unit of Salamanca, and the protocol/registration dates are provided. The manuscript should clarify the exact recruitment and data collection period if available.
Methods	6a	Give the eligibility criteria, and the sources and methods of selection of participants.	Methods, 2.2 Study Population	Participants were recruited by consecutive sampling from the urban health area of Salamanca. Inclusion criteria were age 18-34 years and signed informed consent; exclusion criteria included terminal situation and inability to attend the research unit.
Methods	6b	For matched studies, give matching criteria and number of exposed and unexposed.	Not applicable	This was not a matched study.
Methods	6c	For cross-sectional studies, if applicable, describe analytical eligibility and final analytical sample.	Methods, 2.2 Study Population	The manuscript states that 501 individuals were recruited and 496 with complete MEDAS, EDAS-18, CIUS and CERV questionnaires were

Section/topic	Item	STROBE recommendation	Reported in manuscript	Comments for this manuscript
				included in the analysis.
Methods	7	Clearly define all outcomes, exposures, predictors, potential confounders and effect modifiers. Give diagnostic criteria, if applicable.	Methods, 2.4 Variables and Measurement Instruments; 2.5 Statistical Analysis	The main outcome was MEDAS-14 score and individual MEDAS criteria; predictors were EDAS-18, CIUS, CERV and global digital addiction index; covariates included age, sex, BMI, smoking, alcohol and physical activity. Sex interactions were assessed.
Methods	8	For each variable of interest, give sources of data and details of methods of assessment or measurement. Describe comparability of assessment methods if there is more than one group.	Methods, 2.4.1-2.4.4	Age/sex, alcohol, smoking, IPAQ-SF physical activity, MEDAS-14, EDAS-18, CIUS, CERV and the global digital addiction index are described, including scoring ranges and interpretation.
Methods	9	Describe any efforts to address potential sources of bias.	Methods, 2.4; Discussion, Limitations	Measurements were standardized, performed by trained researchers and quality checked by an independent investigator. The Discussion acknowledges recall bias, social desirability, self-reporting and residual confounding. A dedicated bias subsection could further strengthen reporting.
Methods	10	Explain how the study size was arrived at.	Methods, 2.3 Sample Size Calculation	Sample size was calculated using

Section/topic	Item	STROBE recommendation	Reported in manuscript	Comments for this manuscript
				GRANMO 7.2 based on MEDAS score, alpha 0.05, power >0.80, group sizes 339 and 157, detectable difference 0.54 and SD 2.01.
Methods	11	Explain how quantitative variables were handled in the analyses. If applicable, describe which groupings were chosen and why.	Methods, 2.3, 2.4.3, 2.5	MEDAS and digital addiction metrics were treated as continuous variables for correlation and linear regression; MEDAS adherence was also categorized into low-moderate and high adherence. Individual MEDAS items were analyzed as dichotomous outcomes.
Methods	12a	Describe all statistical methods, including those used to control for confounding.	Methods, 2.5 Statistical Analysis	The manuscript describes t tests, chi-square tests, Pearson correlations, hierarchical multivariable linear regression, VIF diagnostics, sex-interaction terms and binary logistic regression adjusted according to Model 2.
Methods	12b	Describe any methods used to examine subgroups and interactions.	Methods, 2.5 Statistical Analysis; Results	Sex x addiction score multiplicative interaction terms were calculated and introduced into final hierarchical regression blocks. Sex-stratified analyses were planned only if interactions were significant.
Methods	12c	Explain how missing data were addressed.	Methods/Results	The analytic sample was restricted to

Section/topic	Item	STROBE recommendation	Reported in manuscript	Comments for this manuscript
				participants with complete MEDAS, EDAS-18, CIUS and CERV questionnaires. The manuscript should explicitly state whether complete-case analysis was used for all models and quantify missing data for covariates if applicable.
Methods	12d	For cross-sectional studies, describe analytical methods taking account of sampling strategy, if applicable.	Methods, 2.2 and 2.5	Participants were recruited using consecutive sampling; standard regression and comparative methods were applied. No complex sampling design was described.
Methods	12e	Describe any sensitivity analyses.	Methods/Results	No formal sensitivity analyses are described. If none were performed, this may be stated explicitly.
Results	13a	Report numbers of individuals at each stage of study: potentially eligible, examined for eligibility, confirmed eligible, included, completing follow-up, and analysed.	Methods, 2.2; Results	The manuscript reports 501 recruited participants and 496 participants included in the analytic sample after complete questionnaire availability.
Results	13b	Give reasons for non-participation at each stage.	Methods/Results	The final analytic sample is reported, but reasons for excluding the 5 recruited participants not included in the final analysis should be specified if available.

Section/topic	Item	STROBE recommendation	Reported in manuscript	Comments for this manuscript
Results	13c	Consider use of a flow diagram.	Not applicable / optional	A flow diagram is not included. It is optional but could improve clarity by showing recruitment and analytic sample derivation.
Results	14a	Give characteristics of study participants and information on exposures and potential confounders.	Results, Table 1 and Table 2	Tables 1 and 2 describe sociodemographic, anthropometric, lifestyle and digital addiction characteristics by MedDiet adherence and by sex.
Results	14b	Indicate number of participants with missing data for each variable of interest.	Results/Tables	Tables present group totals but do not consistently report variable-specific missing data. The manuscript states a complete questionnaire sample of 496; covariate-specific missingness should be added if present.
Results	14c	For cohort studies, summarize follow-up time.	Not applicable	Cross-sectional study; no follow-up time.
Results	15	Report numbers of outcome events or summary measures.	Results, Tables 1-2 and Figures 1-5	Summary measures are provided for MEDAS score, EDAS-18, CIUS, CERV and global digital addiction score. Logistic models report ORs for each individual MEDAS criterion.
Results	16a	Give unadjusted and, if applicable, confounder-adjusted estimates and their	Results, Figures 3-5; Methods, 2.5	Figures 3-5 provide adjusted beta coefficients or ORs with 95% CIs. The

Section/topic	Item	STROBE recommendation	Reported in manuscript	Comments for this manuscript
		precision. Make clear which confounders were adjusted for and why included.		adjustment structure is described as age/sex and fully adjusted models including BMI, smoking, alcohol and physical activity.
Results	16b	Report category boundaries when continuous variables were categorized.	Methods, 2.4.3; Tables	MEDAS adherence categories are defined as low-moderate and high adherence according to MEDAS cut-off criteria; individual MEDAS item compliance is treated as dichotomous.
Results	16c	If relevant, consider translating estimates of relative risk into absolute risk for a meaningful time period.	Not applicable	The study is cross-sectional and uses linear regression and logistic regression estimates; absolute risk over time is not applicable.
Results	17	Report other analyses done, such as subgroup analyses, interactions and sensitivity analyses.	Results, sex-interaction paragraph; Figures 4-5	Sex-interaction analyses are reported with p-values for EDAS-18, CIUS, CERV and the global digital addiction construct. Item-level logistic regression analyses are also presented.
Discussion	18	Summarize key results with reference to study objectives.	Discussion and Conclusions	The Discussion summarizes that smartphone dependence is inversely associated with MEDAS-14, CIUS shows a different/positive pattern, and CERV/global digital addiction are not consistently

Section/topic	Item	STROBE recommendation	Reported in manuscript	Comments for this manuscript
				associated with overall diet quality.
Discussion	19	Discuss limitations, taking into account sources of potential bias or imprecision. Discuss both direction and magnitude of any potential bias.	Discussion, Limitations	Limitations include cross-sectional design, self-reported questionnaires, recall and social desirability bias, construct differences between scales, lack of objective screen-time/app-use data, residual confounding and limited power for stratified analyses.
Discussion	20	Give a cautious overall interpretation of results considering objectives, limitations, multiplicity of analyses, results from similar studies and other relevant evidence.	Discussion	The manuscript compares findings with previous literature and interprets the CIUS association cautiously, emphasizing heterogeneity of digital phenotypes rather than a homogeneous screen-addiction construct.
Discussion	21	Discuss the generalisability/external validity of the study results.	Discussion, Limitations/Implications	The manuscript discusses implications for students/young adults, but external validity could be strengthened by explicitly noting that the sample comes from one urban area in Salamanca and may not fully represent all young adults.
Other information	22	Give the source of funding and the role of the funders for the present study and, if applicable, for the	Funding section	Funding sources include ISCIII/RICAPPS, IBSAL, Government of Castile and Leon

Section/topic	Item	STROBE recommendation	Reported in manuscript	Comments for this manuscript
		original study on which the present article is based.		research projects and the semFYC doctoral thesis grant. The role of funders is not explicitly stated; add if required by journal.

Abbreviations: BMI, body mass index; CERV, Questionnaire of Experiences Related to Video Games; CIUS, Compulsive Internet Use Scale; EDAS-18, Smartphone Dependence and Addiction Scale; MEDAS, Mediterranean Diet Adherence Screener; OR, odds ratio; STROBE, Strengthening the Reporting of Observational Studies in Epidemiology; VIF, variance inflation factor.

Table S2. Criteria for Efficiency and Scoring of the 14-item Mediterranean Diet Adherence Screen (MEDAS).

Item	Dietary Question / Component	Criteria for 1 Point (Healthy Habit)	Criteria for 0 Points
1	Use of olive oil as main cooking fat	Yes (Preference for olive oil)	No (Use of other fats or oils)
2	Daily olive oil consumption	≥ 4 tables poons/day	< 4 tables poons/day
3	Daily vegetable consumption	≥ 2 servings/day (≥1 portion raw/salad)	< 2 servings/day
4	Daily fruit consumption	>3 pieces/day	< 3 pieces/day
5	Daily red/processed meat consumption	< 1 serving/day	≥ 1 serving/day
6	Daily butter, margarine, or cream consumption	< 1 serving/day	≥ 1 serving/day
7	Daily sweet/sugar-sweetened beverage consumption	< 1 drink/day	≥ 1 drink/day
8	Weekly wine consumption	≥ 7 glasses/week	< 7 glasses/week
9	Weekly legume consumption	≥ 3 servings/week	< 3 servings/week
10	Weekly fish or shellfish consumption	≥ 3 servings/week	< 3 servings/week
11	Weekly commercial sweets or pastry consumption	< 3 times/week	≥ 3 times/week
12	Weekly nuts consumption	≥ 3 servings/week	< 3 servings/week
13	Preference for white meat over red meat	Yes (Preference for chicken, turkey, or rabbit)	No (Preference for beef, pork, or lamb)
14	Weekly consumption of dishes seasoned with *sofrito*	≥ 2 times/week	< 2 times/week

Note: Sofrito is defined as a traditional sauce made by simmering tomato, garlic, onion, and herbs in extra-virgin olive oil.
